# Everybody's Page

**Published:** 1891-02-07

**Authors:** 


### Everybody's Page.

Dr. Mead, who at one time was the greatest of all the London doctors, was assailed in a pamphlet by Dr. Woodward, Professor of Physic in Gresham College. The doctors met and a fight ensued with swords. Dr. Mead having disarmed his adversary called upon him to beg for life. "Never," said Woodward, "never, till I am your patient."

Tooke used to say that Porson would drink anything, even ink rather than not drink at all. He was sitting with a gentleman after dinner in the chambers of a mutual friend, a Templar, who was then ill and confined to bed. A servant came into the room sent thither by his master for a bottle of embrocation which was on the chimney-piece, "I drank it an hour ago," said Porson.

When so much arable land had been thrown into pasture it was feared that much of the latter would be idle or unproductive, or, in other words, become waste, owing to an insufficiency of animals to stock the increased area of the pasture. But a ray of hope for the future of the British farmer is shed by the agricultural returns prepared by Major Craigie, which show that at last this insufficiency has been made up, and that the lands are fully stocked.

"No one now denies the advantages of free libraries." So writes Sir John Lubbock, and the statement is probably true if modified by the addition of the words, "if put to a proper use." The returns of some of the public libraries give strong evidence that the effect of their establishment has not been all that was desired, or is desirable. The better class of books are seldom asked for, but the trash of which this latter half-century has been so remarkably prolific, is only too often eagerly sought after. No one can deny the advantages of a library to working men in our towns, whose lives are of necessity monotonous, if they know how to make good use of it. But it seems Utopian to hope that a better taste can be educated amongst the frequenters of free libraries, so long as the world is flooded with the wretched stuff that is dignified with the name of literature. In order to reap the full benefit to be gleaned from a library, a man "must know how to read." As Richard de Bury, Bishop of Durham, wrote: "The library, therefore, of wisdom is more precious than all riches, and nothing that can be wished for is worthy to be compared with it. Whosoever, therefore, acknowledges himself to be a zealous follower of truth, of happiness, of wisdom, of science, or even of faith, must of necessity make himself a lover of books."

A writer in the *Journal D'Hygiane* takes a somewhat sombre view of society at the present time. It is the fashion to decry everything; to put no faith in honourable sentiments; to look for a sordid motive as underlying every good action. That the present generation of fashionable society is supremely egoistic and totally insensible to everything that is not money or pleasure is probably the not wholly incorrect view of many, though it sounds a somewhat sweeping assertion.

According to Dr. W. Francken, of the University of Amsterdam, the atmosphere on the coast of Holland possesses in a marked degree the qualities of extreme density, constant temperature, purity, moisture, and wealth of saline matter, and, notwithstanding what German authorities have to say, seabaths can be taken with much greater benefit at Schweningen, and other seaside places in the Netherlands, than at that great resort for bathers, Heligoland.

Such weather as we have been experiencing during the past eight weeks is not only the cause of many sad deaths from exposure, but indirectly from asphyxiation as well. It is only too common to hear of deaths resulting from the inhalation of fumes emanating from coal or coke fires in bedrooms. So long as bed-rooms without fire-places or proper ventilation remain uncondemned as not fit for human habitation, we shall continue to hear of pails of coke being left burning in rooms during the night, with fatal results which should and so easily could be made impossible.

Those who have had the misfortune to find it necessary to travel in England during the present winter, must have pondered, probably with a feeling of hopeless resignation, upon the remarkable absence of progress made by all the railway companies in their efforts to warm the compartments of their trains. The old-fashioned footwarmer, which is such a splendid producer of chilblains, and which is a stumbling block to every fresh traveller as he enters the carriage, holds its own with a tenacity truly British. It seems hopeless to expect the introduction of any improved system of constant and regular warming by means of a current of hot air or water supplied from the engine. The resulting outlay might mean a slightly diminished dividend for one year, and it would be so hard that the comfort of the public should be allowed to interfere with that of the shareholders.

